# Influence of Biotic and Abiotic Elicitors on Rosmarinic Acid Accumulation in Hairy Root Cultures of *Dracocephalum kotschyi* Boiss.

**DOI:** 10.3390/plants14172809

**Published:** 2025-09-08

**Authors:** Hoda Sadat Kiani, Manijeh Sabokdast, Beata Dedicova

**Affiliations:** 1 Department of Agronomy and Plant Breeding, College of Agriculture and Natural Resources, University of Tehran, P.O. Box 4111, Karaj 31587-11167, Iran; h.kiani@ut.ac.ir; 2 Department of Plant Breeding, Swedish University of Agricultural Sciences (SLU) Alnarp, Sundsvägen 10, P.O. Box 190, SE 234 22 Lomma, Sweden

**Keywords:** *D. kotschyi*, hairy roots, RA, antioxidant activity, yeast extract, TiO_2_, metabolic stimulation

## Abstract

*Dracocephalum kotschyi* Boiss., an endangered and endemic medicinal plant of Iran belonging to the Lamiaceae family, is a valuable source of methoxylated flavonoids with proven anticancer activity. In this study, hairy roots were effectively induced from two-week-old hypocotyl explants using *Agrobacterium rhizogenes strain* ATCC 15834. Biomass optimization showed that maximum seedling growth occurred in ¼-strength MS medium, while the highest hairy root induction (76.55%) was achieved in ½-strength MS medium supplemented with 1 mM L-arginine. Root induction varied significantly with explant type and age, with the lowest response (14.66%) observed in five-week-old leaf explants. To increase rosmarinic acid (RA) accumulation, transgenic hairy roots were treated with yeast extract (200 mg L^−1^) as a biotic elicitor and titanium dioxide (TiO_2_) nanoparticles (200 and 400 mg L^−1^) as abiotic elicitors for 24 and 48 h. HPLC analysis revealed that treatment with yeast extract (200 mg L^−1^ for 48 h) resulted in the highest accumulation of rosmarinic acid (5.65 mg g^−1^ DW), corresponding to a 26% increase over the control, accompanied by a substantial enhancement of antioxidant activity (63.4%). Yeast extract application also elevated total protein content and glutathione peroxidase (GPX) activity, while markedly suppressing ascorbate peroxidase (APX) and polyphenol oxidase (PPO) activities. In contrast, TiO_2_ nanoparticles, particularly at a concentration of 400 mg L^−1^ for 48 h, augmented APX and PPO activities, indicating the induction of oxidative stress. These findings demonstrate that yeast extract and low concentrations of TiO_2_ nanoparticles can serve as effective elicitors to enhance rosmarinic acid accumulation in *D. kotschyi* hairy root cultures, providing valuable insights for applications in plant biotechnology.

## 1. Introduction

For centuries, medicinal plants have played a vital role in maintaining human health, providing natural remedies, and serving as the basis for many pharmaceutical drugs used today. Their value stems from their ability to combat a wide range of conditions, from inflammation to cancer, due to the variety of active compounds they contain. Ongoing research into these bioactive substances continues to underscore their significance in contemporary medical practices [1,2].

Zarrin-giah or Dena lemon balm, scientifically known as *Dracocephalum kotschyi* Boiss., is a medicinal and aromatic plant belonging to the Lamiaceae family. It is endemic to the high-altitude mountainous regions of Iran, especially the Zagros and Alborz ranges. Morphologically, it is an annual or perennial herb with simple leaves and bilabiate flowers. Phytochemical studies have identified various bioactive compounds in this species, including methoxylated flavonoids (such as apigenin, luteolin, xanthomicrol), rosmarinic acid, monoterpene glycosides, and phenylpropanoids, which contribute to its documented antioxidant, anti-inflammatory, anticancer, and immunomodulatory activities. [3,4].

Hairy roots, induced by *Agrobacterium rhizogenes* strain ATCC 15834 through rol gene transfer, serve as effective in vitro systems for producing secondary metabolites [5,6]. Due to their genetic stability and high metabolic activity, they are widely used in biotechnology and the production of pharmaceutical compounds [7]. Hairy root cultures offer a promising system for producing rosmarinic acid (RA) because of their hormone-independent growth and increased metabolic flux through phenylpropanoid pathways [5,8]. Elicitors, biotic or abiotic agents, enhance metabolite production by activating defense pathways and increasing biosynthetic gene expression, thereby boosting the synthesis of compounds such as rosmarinic acid [9,10].

Rosmarinic acid (RA) is a naturally occurring polyphenolic compound, classified as an ester of caffeic acid and 3,4-dihydroxyphenyllactic acid. It was first isolated from *Rosmarinus officinalis* and is commonly found in medicinal plants within the Lamiaceae family, including *Salvia officinalis*, *Mentha* × *piperita*, *Melissa officinalis*, and *Symphytum officinale* [11,12]. RA is well known for its potent antioxidant properties, reportedly surpassing those of vitamin E [13]. It also exhibits a broad range of pharmacological activities, including anti-inflammatory, antiviral, antibacterial, anticancer, and neuroprotective effects, with potential applications in treating asthma, allergies, and neurodegenerative diseases such as Alzheimer’s [14,15]. Recent advancements in plant biotechnology have significantly enhanced the production of rosmarinic acid through the establishment of hairy root cultures. Despite these developments, challenges persist in optimizing elicitation strategies and improving overall yield, underscoring the need for further research in this area [16,17,18].

The main goal of this study is to boost the biosynthesis of rosmarinic acid, a key secondary metabolite known for its strong antioxidant and therapeutic effects, by inducing hairy root formation using two elicitors: a biotic elicitor (yeast extract) and an abiotic elicitor (titanium dioxide nanoparticles, TiO_2_ NPs). This approach lays the groundwork for exploring potential uses in natural product-based drug development, while the antioxidant activity of the treated samples is measured to evaluate their functional properties.

## 2. Results

### 2.1. Short-Term Moist Stratification Promotes Dormancy Release in D. kotschyi Seeds

**Figure 1:**
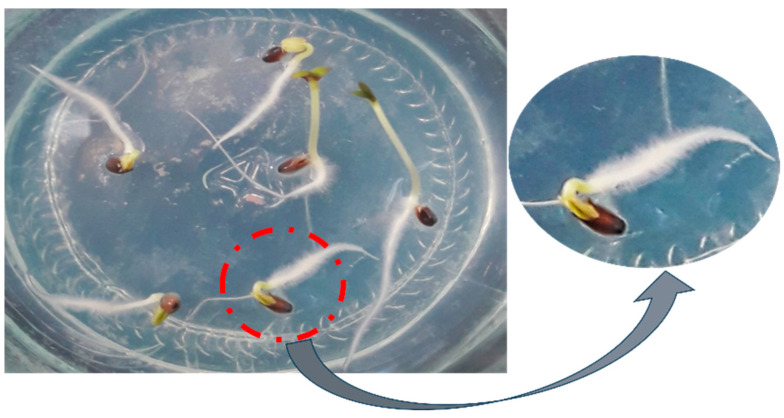
Germination of *D. kotschyi* seeds. 100% germination was achieved after 7 days following the placement of surface-sterilized seeds on ¼-strength MS medium.

To overcome seed dormancy in *D. kotschyi*, seeds were subjected to moist cold stratification at 5 ± 1 °C for 48 h. Following this treatment, the seeds were transferred to ¼-strength MS medium and maintained in a growth chamber under controlled conditions (25 ± 2 °C; 16/8 h light/dark photoperiod). Within six days, radicle emergence was observed in 99–100% of the samples (Figure 1). A chi-square (χ^2^) test confirmed that the increase in germination was statistically significant compared to the untreated control group, which exhibited no germination during the same period (*p* < 0.01). Germination indices also showed notable improvement, with a mean germination rate (GR) of 23.8 ± 1.2 seeds per day and a time to 50% germination (T_50_) of 2.1 ± 0.3 days.

**Figure 2:**
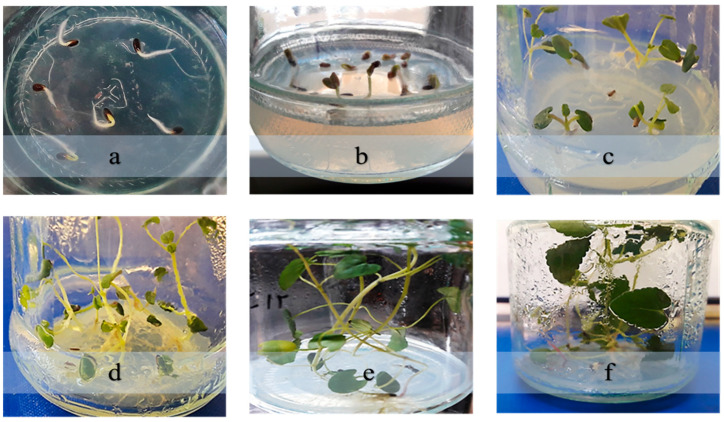
Germination and seedling development of *D. kotschyi* under in vitro conditions. (**a**) Seed germination occurs five days after the seeds are placed on culture medium. (**b**) Emergence of the shoot and cotyledons in a one-week-old seedling. (**c**) Two-week-old seedling. (**d**) Three-week-old seedling. (**e**) Four-week-old seedling. (**f**) Five-week-old seedling.

Regarding explant age, a marked decline in responsiveness was observed with increasing age: two-week-old hypocotyls had an induction rate of 56%, while five-week-old samples exhibited only 14.66% (Figure 2).

### 2.2. The Effect of Explant Type and Age on Hairy Root Induction

**Figure 3:**
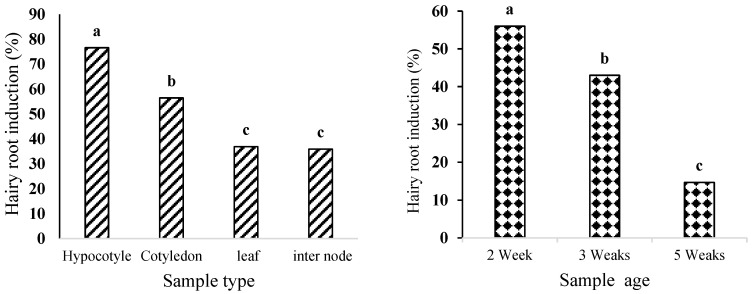
The effect of hypocotyl microspecimen age and microspecimen type on the percentage of hairy root induction in *D. kotschyi*. Means with the same letter did not differ significantly (*p* > 0.05) based on Duncan’s multiple range test.

**Figure 4:**
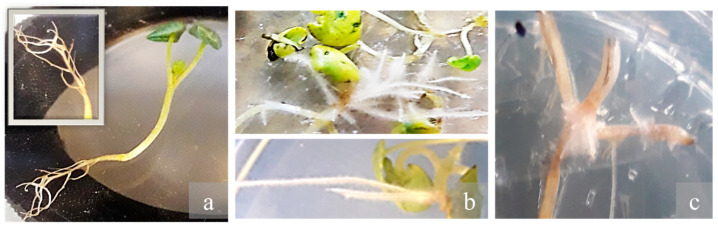
Hairy root induction in different explants of *D. kotschyi* under in vitro conditions, and emergence of hairy roots two to three weeks after bacterial inoculation. (**a**) Control: standard, non-transformed roots. (**b**) Emergence and induction of hairy roots in hypocotyl explants. (**c**) Emergence of hairy roots in internode explants.

Hairy root formation was successfully induced in *D. kotschyi* explants following inoculation with *A. rhizogenes* strain ATCC 15834. Hairy roots emerged within 10 days and continued to elongate for up to four weeks. No root development occurred in the non-inoculated controls. Analysis of variance (Table S1) demonstrated that both explant type and age had a statistically significant effect on induction efficiency (*p* < 0.01; Figure 3). Two-week-old hypocotyls exhibited the highest induction rate (76.5%), whereas internodes showed the lowest (20%) (Figure 4).

### 2.3. The Effect of L-Arginine on Hairy Root Induction

**Figure 5:**
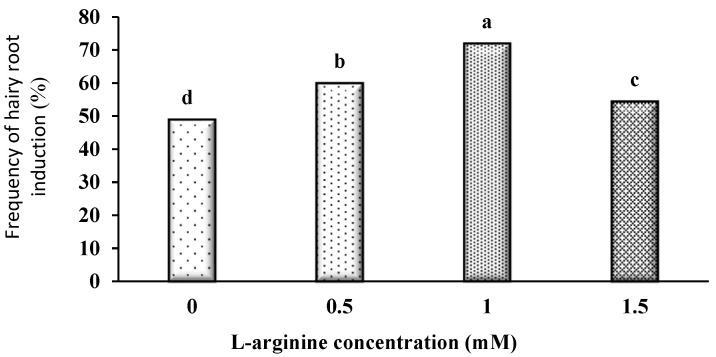
Comparison of the average concentrations of different amino acids, including L-arginine, on hairy root induction. Identical letters indicate no significant difference at the 5% probability level with Duncan’s test.

The incorporation of L-arginine into the co-cultivation medium significantly enhanced the induction of hairy roots in *D. kotschyi* (Table S2). Specifically, treatment with 1 mM L-arginine led to an approximately 23% increase in root induction frequency compared to the untreated control. This concentration also resulted in the lowest incidence of necrosis among wounded explants. Although increasing the concentration up to 1 mM improved the induction responses, further elevation to 1.5 mM caused a decline in hairy root formation (Figure 5).

### 2.4. Hairy Root Growth and Development

**Figure 6:**
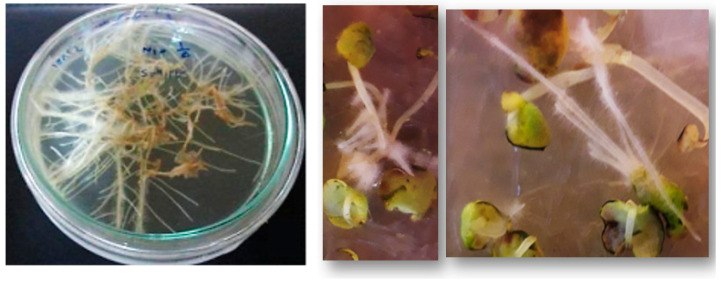
Emergence of hairy roots at wounding sites on leaf explants and hypocotyl, and maximum hairy root proliferation in *D. kotschyi*.

**Figure 7:**
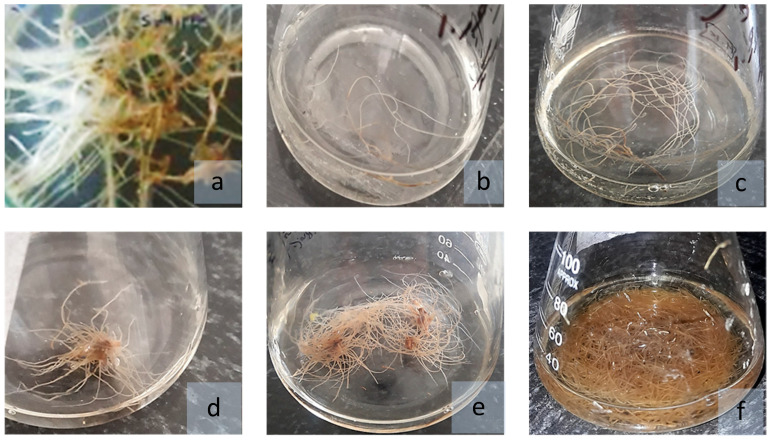
Growth progression of hairy roots of *D. kotschyi* in MS medium. [(**a**) Twenty-day-old hairy roots on solid MS medium; (**b**–**e**) Hairy roots cultured in liquid ½ MS medium at 10, 21, 30, and 45 days, respectively; (**f**) Two-month-old brownish hairy roots grown in liquid MS medium].

Inoculation of *D. kotschyi* with *A. rhizogenes* strain ATCC 15834 successfully induced hairy root formation at wounded internodal and leaf vein regions. In some explants, root initiation occurred directly, while in others it was preceded by callus formation (Figure 6). The resulting roots reached full development within 20 days on solid, full-strength MS medium. Upon transfer to hormone-free liquid ½ MS medium supplemented with L-arginine, they proliferated extensively over the next two months (Figure 7).

### 2.5. Molecular Verification of the Transgenic Nature of Hairy Roots

**Figure 8:**
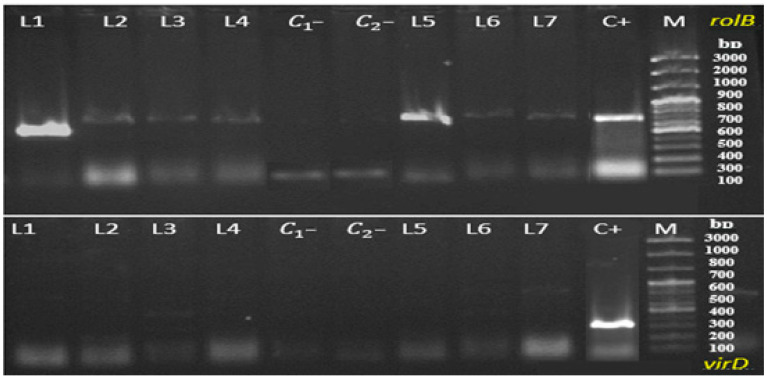
PCR analysis for confirmation of *rol*B gene presence and *vir*D gene absence in transgenic hairy roots of *D. kotschyi*. [M: DNA ladder; C+: *A. rhizogenes* strain ATCC 15834 as positive control; C_1_–: no-template control (PCR reaction without DNA); C_2_–: non-transformed root sample as negative control. L1–L7: Hairy root lines induced from hypocotyl explants by *A. rhizogenes* strain 15834.

Genomic DNA was extracted from *D. kotschyi* hairy root lines using a modified CTAB protocol. The quality and purity of the extracted DNA were confirmed by spectrophotometric analysis, with absorbance ratios at 260/280 nm and 260/230 nm close to 2.0, indicating high-purity DNA suitable for downstream molecular applications [19]. PCR analysis using *rol*B-specific primers successfully amplified the expected 780 bp fragment in all hairy root samples, confirming the stable integration of T-DNA into the plant genome. Conversely, amplification of the *virD* gene (338 bp), a marker of Agrobacterium contamination, was not detected in any of the samples, verifying the absence of residual bacterial DNA. While all hairy root lines tested positive for the *rol*B gene, lines L1 and L5 exhibited distinctly sharper and more consistent amplification bands (Figure 8). Consequently, these two lines were selected for subsequent biochemical and phytochemical analyses.

### 2.6. Effects of Biotic and Abiotic Elicitors on Biochemical Traits in D. kotschyi Hairy Roots

The application of yeast extract (200 mg L^−1^) and TiO_2_-NPs (200 and 400 mg L^−1^) significantly influenced the biochemical responses of *D. kotschyi* hairy roots, with effects dependent on the type of elicitor, its concentration, and the duration of exposure (Table S3).

#### Determination of Total Protein Concentration

**Figure 9:**
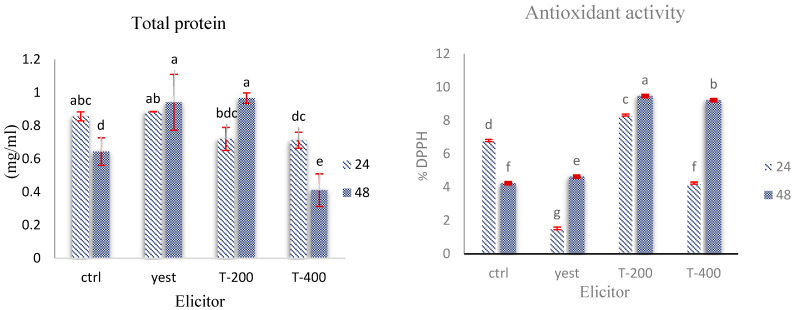
Mean comparison of the interactive effects of elicitor concentration and exposure time (TiO_2_ NPs and yeast extract) on total protein content and overall antioxidant activity (DPPH assay) in hairy roots of *D. kotschyi*. Treatments include: control (Ctrl), yeast extract (Yeast), TiO_2_ NPs at 200 mg L^−1^ (T-200), and 400 mg L^−1^ (T-400). Bars with the same letter are not significantly different at *p* < 0.05 based on Duncan’s multiple range test.

In *kotschyi* hairy roots, suspension treatment with yeast extract (200 mg L^−1^) and TiO_2_ NPs (200 and 400 mg L^−1^) for 24 and 48 h induced significant changes in total protein content (*p* < 0.01, Duncan’s test). The highest protein accumulation was observed in the yeast extract treatment and the 200 mg L^−1^ TiO_2_ treatment after 48 h of incubation. In contrast, the 400 mg L^−1^ TiO_2_ treatment, particularly after 48 h, resulted in a ≈reduction of approximately 30% in protein levels compared to the control (Figure 9).

### 2.7. Assessment of Antioxidant Enzyme Dynamics in D. kotschyi Hairy Roots Under TiO_2_ NPs and Yeast Extract Treatments

Treatment of *D. kotschyi* hairy roots with TiO_2_-NPs and yeast extract resulted in a marked increase in antioxidant enzyme activity at 24 h post-treatment, followed by a decline at 48 h. This temporal response suggests that the elicitors triggered a short-lived oxidative reaction in the root tissues.

#### 2.7.1. GPX Activity

**Figure 10:**
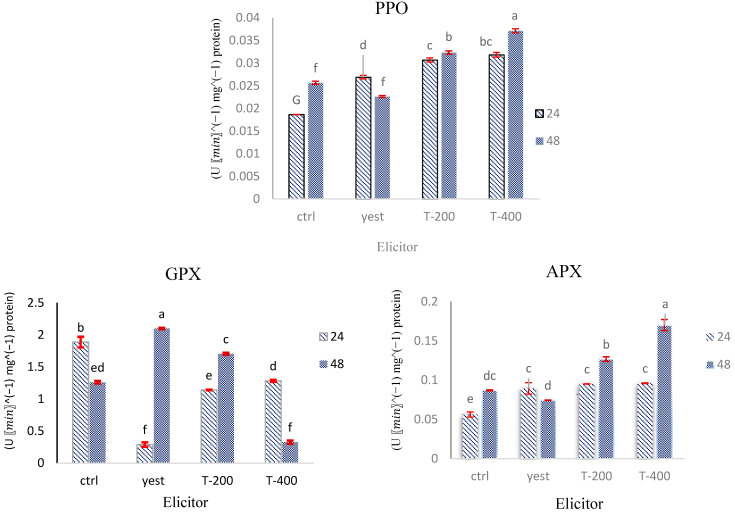
Comparison of the Mean Interaction Effects of Concentration and Treatment Duration with TiO_2_ NPs and Yeast Extract on the Activities of GPX, APX, and PPO in Hairy Roots of *D. kotschyi*.Control (Ctrl), Yeast Extract (Yeast), TiO_2_ NPs at 200 mg L^−1^ (T-200), and 400 mg L^−1^ (T-400). Identical letters indicate no statistically significant differences among means at the 0.05 probability level (*p* < 0.05).

GPX activity in *D. kotschyi* hairy roots was significantly influenced by the interaction between elicitor concentration and exposure duration. Treatment with yeast extract caused a 65.8% reduction in GPX activity at 24 h, followed by a compensatory increase of 66.6% at 48 h. Application of 200 mg L^−1^ TiO_2_-NPs for 48 h resulted in a 71% increase in GPX activity, whereas a higher dose of 400 mg L^−1^ under the same conditions led to suppression of enzyme activity (Figure 10).

#### 2.7.2. APX Activity

APX activity in *D. kotschyi* hairy roots was significantly affected by the type of elicitor, treatment duration, and their interaction (*p* < 0.01). The highest induction (95.06%) occurred after treatment with 400 mg L-1 titanium dioxide (TiO_2_) nanoparticles for 48 h. Both concentrations of TiO2-NPs caused a modest increase (~7%) in APX activity at 24 h. Yeast extract treatment increased APX activity by 58.61% at 24 h but decreased it by 14.67% below control levels after 48 h (Figure 10).

#### 2.7.3. PPO Activity

The activity of PPO in *D. kotschyi* hairy roots was significantly affected (*p* < 0.01) by both biotic (yeast extract) and abiotic (TiO_2_-NPs) elicitors, as shown in Table S3. The highest PPO activity was observed after treatment with 400 mg L^−1^ TiO_2_-NPs for 48 h, whereas exposure to yeast extract over the same period resulted in a moderate decrease (12.06%) compared to the control. Although there was an overall upward trend in PPO activity across treatments, the extent of variation was less noticeable compared to other antioxidant enzymes (Figure 10).

### 2.8. Effects of Biotic and Abiotic Elicitors on Rosmarinic Acid Accumulation in Hairy Roots of D. kotschyi

**Figure 11:**
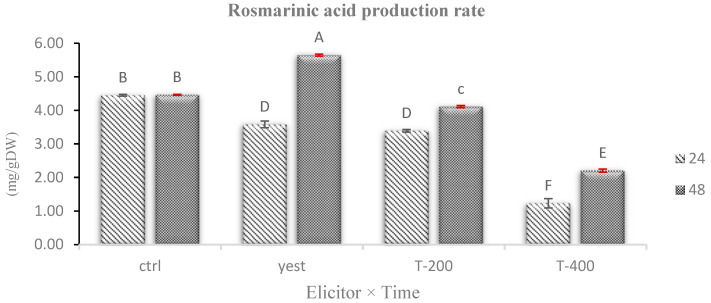
Comparison of the mean effects of the interaction between concentration and treatment duration of TiO_2_ NPs and yeast extract on rosmarinic acid content in hairy roots of *D. kotschyi*. Treatments include control (Ctrl), yeast extract (Yeast), TiO_2_ NPs at 200 mg L^−1^ (T-200), and 400 mg L^−1^ (T-400). Means followed by the same letters are not significantly different at *p* < 0.05.

The results of this study showed that treating *D. kotschyi* hairy roots with biotic (yeast extract) and abiotic (TiO_2_ NPs) elicitors caused significant changes in rosmarinic acid levels (*p* < 0.01; Table S4). The highest buildup of this phenolic compound was observed after treatment with yeast extract at 200 mg L^−1^ for 48 h, resulting in approximately a 1.26-fold increase compared to the control. In contrast, most TiO_2_ NP treatments significantly decreased rosmarinic acid levels, with the lowest amount recorded after exposure to 400 mg L^−1^ for 24 h (Figure 11).

## 3. Discussion

These findings suggest that a brief period of moist chilling (48 h) is sufficient to break physical or physiological dormancy in *D. kotschyi* seeds, eliminating the need for mechanical scarification or other invasive methods. This supports earlier reports showing that cold stratification at 5 °C for 24 to 72 h significantly boosts both germination percentage and rate in this species [20,21]. Additionally, the beneficial effect of low-temperature moist stratification on seed germination has been confirmed in other temperate species, such as *Abies pindrow* and *Picea smithiana*, further highlighting the vital role of cold-induced dormancy alleviation across various taxonomic groups [22]

Regarding explant age, two-week-old hypocotyls showed a 56% induction rate, while five-week-old samples displayed only a 14.66% induction rate (Figure 2). This difference likely stems from the higher metabolic activity and meristematic potential of younger cells [23]. Hypocotyl tissues, due to their active cell populations and greater contact surface with the bacterium, demonstrated greater competence for foreign gene uptake. In contrast, internodal segments, which have fewer dividing cells and a more compact structure, exhibit lower transformation efficiency [24]. These results align with previous reports in *Cannabis sativa* L. [24] and *Fragaria vesca* [25]. Overall, the two-week-old hypocotyl is identified as the best explant for efficient hairy root induction in *D. kotschyi*.

The physiological effects of arginine likely stem from its involvement in essential biosynthetic pathways, including those for nitric oxide, polyamines, and proline, as well as its role in reducing oxidative stress [26,27]. These findings are consistent with recent reports emphasizing the benefits of organic nitrogen sources in enhancing regeneration efficiency, decreasing tissue necrosis, and improving genetic stability in *A. rhizogenes*-mediated transformation systems [28,29].

The TL-DNA region of the Ri plasmid in this bacterial strain contains four *rol* genes, *rol*A, *rol*B, *rol*C, and *rol*D, that are essential for rhizogenic morphogenesis. Notably, *rol*A, *rol*B, and *rol*C alone are sufficient to induce hairy roots. These genes increase the sensitivity of plant cells to endogenous auxins without changing their internal levels, thereby triggering the root differentiation process. Additionally, the *rol*D gene, which encodes the enzyme ornithine cyclodeaminase, promotes root growth by influencing amino acid metabolism related to cell proliferation [30,31,32].

The molecular analysis provides clear evidence supporting the successful genetic transformation of *D. kotschyi* hairy root cultures. The consistent amplification of the *rol*B gene across all tested lines demonstrates the stable integration of T-DNA and confirms the transgenic nature of the root tissues [33]. Additionally, the absence of *vir*D gene amplification confirms the effective removal of *Agrobacterium* after co-cultivation, which is essential for ensuring genetic stability and experimental reliability [34]. These findings strengthen the effectiveness of the transformation protocol used and highlight its potential for creating genetically stable hairy root lines suitable for downstream biotechnological applications, such as elicitor treatments and the production of secondary metabolites.

The observed differences in total protein content across the TiO_2_ nanoparticle treatments show that protein accumulation in *D. kotschyi* hairy roots depends on nanoparticle concentration. Specifically, treatment with 200 mg L^−1^ TiO_2_ resulted in a significant increase in total protein levels after 48 h, indicating that this lower concentration effectively acts as an elicitor, likely by stimulating protein biosynthesis pathways. In contrast, exposure to 400 mg L^−1^ TiO_2_, especially over more extended periods, led to a noticeable decline in protein content, suggesting that higher concentrations may inhibit biosynthetic processes or promote protein breakdown, possibly through oxidative stress [35]. The increase in protein content after yeast extract treatment may be linked to the activation of transcription factors involved in the phenylpropanoid pathway and antioxidant defenses, an effect recently observed in callus cultures of *Limonium algarvense*, where 50–100 mg L^−1^ yeast extract significantly boosted both soluble protein and total phenolic content [36]. Overall, these findings highlight the significant influence of elicitor type and concentration on boosting total protein content in hairy root cultures. Moderate levels of TiO_2_ and yeast extract seem to promote protein biosynthesis by activating metabolic and defense mechanisms. Conversely, higher doses of TiO_2_ appear to be inhibitory, likely due to the cytotoxic effects of reactive oxygen species (ROS).

The observed trend in antioxidant activity, where 200 mg L^−1^ TiO_2_ at 48 h produced the highest radical-scavenging capacity, and 400 mg L^−1^ showed slightly lower activity, highlights a concentration-dependent response to TiO_2_ nanoparticles. This pattern agrees with previous studies that report lower concentrations of TiO_2_ can stimulate antioxidant defense mechanisms and phenolic biosynthesis. In contrast, higher concentrations may suppress these pathways due to increased production of reactive oxygen species (ROS) [37,38]. Additionally, the biotic elicitor yeast extract also enhanced antioxidant activity compared to the control at both time points, likely by activating the phenylpropanoid pathway and increasing the accumulation of defense-related compounds. Similar effects have been seen in *Limonium algarvense* callus cultures treated with 50–100 mg L^−1^ yeast extract, which showed elevated levels of both soluble proteins and total phenolics [36]. Overall, these findings suggest that 200 mg L^−1^ TiO_2_ nanoparticles and 200 mg L^−1^ yeast extract are near-optimal doses for maximizing antioxidant capacity in hairy-root cultures without introducing additional stress. Conversely, higher nanoparticle concentrations do not necessarily result in greater effectiveness. These results offer a practical framework for optimizing the production of valuable antioxidative metabolites in hairy root systems. They also support emerging evidence that elicitor-driven antioxidant regulation depends on the dose in plant suspension cultures [39].

The observed temporary increase in antioxidant enzyme activity indicates a rapid yet short-lived activation of the plant’s oxidative defense system, probably caused by mild oxidative stress from elevated levels of reactive oxygen species (ROS). This pattern supports the concept of stress priming, where exposure to sublethal stress prepares plant tissues for more robust metabolic responses. Similar trends have been observed in other medicinal plants exposed to both biotic and abiotic elicitation [35,40], highlighting the potential of such treatments to metabolically prime the hairy root system and enhance the production of secondary metabolites.

The initial decrease and subsequent increase in GPX activity after yeast extract treatment suggests a priming-induced reprogramming of the antioxidant response, aligning with previous findings in *Arnica montana* in vitro cultures exposed to biotic elicitors [41].The dose-dependent, biphasic response to TiO_2_-NPs further supports the idea that both elicitor strength and exposure duration are critical in shaping the cell’s redox status. Low to moderate doses appear to enhance antioxidant defenses, while higher doses might surpass the cellular threshold, resulting in enzyme inhibition [42]. These findings highlight the intricate regulation of GPX activity in response to external stress and its crucial role in maintaining redox balance during elicitor-induced oxidative stress.

The biphasic response of APX activity under yeast extract treatment likely reflects the dynamics of ROS signaling. The initial stimulation at 24 h may result from temporary increases in hydrogen peroxide (H_2_O_2_), a known inducer of APX gene expression [43]. However, prolonged exposure appears to deplete ROS or trigger feedback inhibition, leading to enzyme downregulation [44]. In contrast, TiO_2_-NPs, especially at a concentration of 400 mg L^−1^ for 48 h, significantly enhance APX activity, consistent with studies highlighting their role in modulating the redox balance and enhancing antioxidant capacity [35,38,42]. These findings emphasize the importance of both elicitor dosage and exposure time in shaping the enzymatic antioxidant response. TiO_2_-NPs at 400 mg L^−1^ show potential for improving oxidative defense mechanisms in hairy root cultures of *D. kotschyi*.

The relatively stable fluctuation in PPO activity may reflect the enzyme’s sensitivity to changes in ionic concentration and osmotic potential within the cellular environment. These findings align with previous studies; for example, Exposure to TiO_2_ nanoparticles at 200 mg L^−1^ has been shown to significantly elevate PPO activity in *Vitex agnus* cactus roots, with recorded levels of 4.23 mg g^−1^ DW compared to 1.38 mg g^−1^ in controls [45,46]. The observed increase in PPO activity appears to be associated with the activation of defense-related pathways, particularly lignification. While both biotic and abiotic elicitors are capable of inducing PPO activity, the magnitude of this response is typically lower than that of key antioxidant enzymes such as APX and GPX. This pattern implies that PPO functions in a complementary capacity within the oxidative defense network of *D. kotschyi* under elicitor exposure, contributing to secondary defense processes rather than serving as a primary enzymatic barrier [47].

The observed decrease in rosmarinic acid at higher nanoparticle concentrations likely results from oxidative stress and the overproduction of reactive oxygen species (ROS). This stress may disrupt the cellular redox balance and inhibit the biosynthesis of secondary metabolites, including those involved in the phenylpropanoid pathway [48,49]. Both past and recent studies have demonstrated that high doses of TiO_2_ NPs can decrease rosmarinic acid production by suppressing key enzymes, such as phenylalanine ammonia-lyase (PAL) and tyrosine aminotransferase (TAT) [50,51]. Additionally, evidence suggests that TiO2 NPs induce cytotoxic effects, damage cellular structures, disrupt defense signaling pathways, and reduce gene expression related to metabolic processes [52,53].

A limitation of this study is that elicitor effects were not assessed in non-transformed roots, and such comparative analyses would be valuable in future research to further clarify the role of genetic transformation in modulating secondary metabolite biosynthesis. Nevertheless, the present findings highlight that the type of elicitor, its concentration, and the duration of treatment are critical factors in successfully activating biosynthetic pathways and enhancing the production of secondary metabolites such as rosmarinic acid in hairy root cultures. Optimizing these parameters can therefore play a pivotal role in the industrial-scale production of pharmaceutically valuable plant-derived compounds.

## 4. Materials and Methods

### 4.1. Plant Material and Culture Conditions

**Figure 12:**
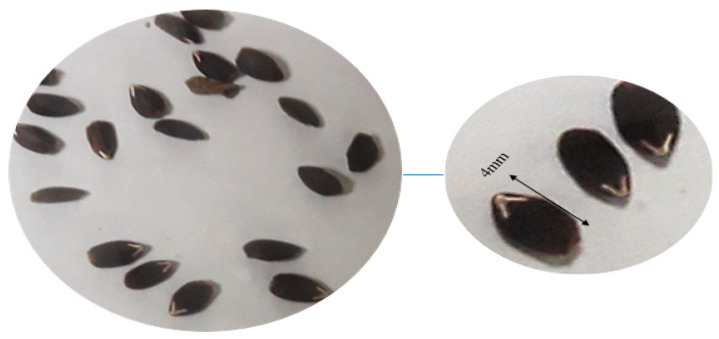
Size, shape, and color characteristics of *D. kotschyi* seeds: seed length approximately 4 mm, width 1.25 mm, and diameter 1.07 mm. Seeds are ovoid in shape, with a prominent convex ventral surface. The apex is lobed, exhibiting a distinct figure-eight-shaped groove, which is more pronounced on the ventral side. The seed coat is dark brown, with an oblique, figure-eight-shaped marking near the apex.

Seeds of *D. kotschyi* were obtained from the Seed and Plant Improvement Institute (SPII, Karaj, Iran) (Figure 12). Freshly harvested, one-year-old seeds were used for all experiments. Surface sterilization was performed under a laminar flow hood as follows: seeds were immersed in 70% ethanol for 8 s, rinsed twice for 2–3 min each with double-sterilized distilled water, treated with 5% sodium hypochlorite for 8 min, and rinsed again twice for 2–3 min with double-sterilized distilled water. This rinse was repeated 2–3 times to ensure complete removal of contaminants and adequate water uptake.

The sterilized seeds were then transferred to one-quarter strength Murashige and Skoog (¼ MS) [54] basal medium, supplemented with 30 g L^−1^ sucrose, 100 mg L^−1^ myo-inositol, and essential micronutrients. The medium pH was adjusted to 5.7–5.8 before autoclaving at 121 °C and 1.5 atm for 30 min. To break seed dormancy, culture vessels containing the inoculated seeds were cold-stratified at 5 °C for 48 h. Germination occurred in a controlled growth chamber maintained at 20–25 °C with 60–70% relative humidity, under a 16-h light/8-h dark photoperiod at a light intensity of 100 µmol m^−2^ s^−1^. Radicle emergence was observed within 7 to 10 days. All experiments were conducted under in vitro conditions using a factorial design arranged in a completely randomized manner with three biological replicates.

### 4.2. Effect of Explant Type and Age on Hairy-Root Induction

The influence of explant type and physiological age on the percentage of hairy root induction was examined using four sources: cotyledon, hypocotyl, leaf plus petiole, and a two-week-old internode from aseptically grown seedlings cultured on hormone-free MS basal medium. To evaluate the effect of explant age, hypocotyl segments were prepared from sterile seedlings at 2, 3, and 5 weeks of age. All treatments were arranged in a completely randomized design with three replicates, each consisting of ten explants. The percentage of explants developing hairy roots was recorded for each treatment.

### 4.3. Bacterial Strain and Culture Conditions

Hairy root induction was performed using *A. rhizogenes* strain ATCC 15834. Bacterial cells were first streaked onto solid LB medium [55] and incubated at 26 °C. A single colony (approximately needle-tip size) was then transferred to liquid LB with rifampicin (50 mg/L) to ensure strain purity. The culture was incubated in darkness at 26 °C on a rotary shaker (180 rpm) for 48 h to produce an overnight suspension suitable for explant inoculation.

### 4.4. Preparation of Co-Cultivation Medium

To identify the best co-cultivation medium for inducing hairy roots, MS medium was supplemented with different concentrations of the amino acid L-arginine. The experiment was set up as a completely randomized design to assess the effect of L-arginine on root induction. The tested concentrations included 0, 0.5, 1.0, and 1.5 mM. Before bacterial inoculation, sterile explants were pre-cultured for 48 h (under a 16-h light/8-h dark photoperiod) on the designated medium.

### 4.5. Explant Inoculation and Bacterial Elimination

Explants were wounded with a scalpel blade dipped in a freshly prepared *A. rhizogenes* suspension (cultured for 16–24 h), then transferred to hormone-free MS or ¼ MS medium [54]. Control explants were wounded with a sterile scalpel without bacterial exposure. Petri dishes were kept in the dark at 24 °C for 72 h, then transferred to a growth chamber under a 16:8 light:dark photoperiod.

To eliminate bacterial contamination, 72 h after inoculation, the explants were briefly dried on sterile filter paper and then transferred with sterile forceps to fresh solid MS medium supplemented with the appropriate concentrations of L-arginine and 500 mg L^−1^ cefotaxime. This process was repeated several times on media containing 300 mg L^−1^ cefotaxime to ensure complete removal of the bacteria. Following root emergence, all Petri dishes were assessed for hairy root traits, including callus induction, rooting percentage, and the number of root branches per explant.

### 4.6. Molecular Confirmation of Transgenic Hairy Roots

#### 4.6.1. Primer Design

Gene-specific primers were designed using Primer3 and validated with Oligo 7 software to amplify a fragment of the *rolB* gene, which was used to verify the transgenic status of the hairy roots, and the *vir*D gene, which served to detect residual *A. rhizogenes* contamination. Primer sequences and characteristics are presented in Table 1.

#### 4.6.2. Genomic and Plasmid DNA Isolation

Genomic DNA was extracted from putative transgenic hairy roots using the protocol described by Khan et al. [56], with minor modifications to optimize yield and purity. To confirm the presence of the Ri plasmid in the bacterial strain used, plasmid DNA was isolated from *A. rhizogenes* ATCC 15834 using a standard method for plasmid isolation. [57]. This preparation served as a positive control in subsequent PCR assays. DNA concentration and purity were assessed spectrophotometrically (A_260_/A_280_), and integrity was verified by agarose-gel electrophoresis before PCR analysis.

#### 4.6.3. PCR Amplification of *rolB* and *virD* Genes

PCR amplification of the *rolB* gene was conducted to confirm the successful genetic transformation of *D. kotschyi* hairy root cultures. In addition, PCR targeting the *virD* gene was performed to verify the complete elimination of *A. rhizogenes* following co-cultivation.

### 4.7. Establishment and Growth of Hairy-Root Cultures

#### 4.7.1. Initiation and Maintenance of Clonal Lines

After confirming transgenicity and verifying the presence of the Ri plasmid, the most vigorous hairy root lines were subcultured on both solid and liquid media to obtain stable clones. Actively growing roots were excised from the solid medium and transferred to hormone-free ½-strength MS medium. [54] liquid medium supplemented with 500 mg L^−1^ cefotaxime. Cultures were kept in darkness on a rotary shaker at 121 rpm and 22–25 °C.

The fastest-growing line derived from the *A. rhizogenes* strain ATCC 15834 was selected based on PCR confirmation and then propagated in liquid culture. After ten weeks, the biomass was cut into ~5 cm fragments and re-inoculated into 40 mL of hormone-free ½-strength MS medium in 100 mL Erlenmeyer flasks. Two weeks after subculturing, the expanded hairy roots were ready for elicitor treatments.

#### 4.7.2. Preparation of Elicitors

The yeast extract elicitor was prepared by dissolving 200 mg of yeast extract powder (Sigma-Aldrich Inc., St. Louis, MO, USA) in 1 L of double-distilled water, followed by sterilization at 121 °C for 30 min. TiO_2_ NPs were prepared separately: precisely weighed amounts of TiO_2_ powder were dispersed in ½-strength MS medium at final concentrations of 200 mg L^−1^ and 400 mg L^−1^ (equivalent to 200 and 400 ppm, respectively), and homogenized using an ultrasonic bath to ensure uniform particle distribution (Table 2). After elicitation with each elicitor separately, cultures were monitored for rooting frequency and the number of root branches per explant.

### 4.8. Determination of Total Protein Content and Antioxidant Enzyme Activities

#### 4.8.1. Preparation of Extraction Buffer and Crude Enzyme Extract

The extraction buffer was prepared according to Pagariya et al. [58], with minor modifications. All glassware and plasticware were autoclaved twice. For each assay, 100 mg of frozen hairy root powder was placed in 2 mL microtubes and homogenized with 2 mL of ice-cold phosphate buffer (pH 7.6) containing 1% (*w*/*v*) polyvinylpyrrolidone (PVP) and 1 mM EDTA. The buffer was prepared one day earlier and stored at 4 °C. The samples were vortexed for 20 s, then incubated on ice for 30 min. They were then centrifuged at 15,000× *g* for 20 min at 4 °C. The supernatant was aliquoted into five pre-labeled 0.2 mL PCR tubes, flash-frozen in liquid nitrogen, and stored at −20 °C. This crude extract was used to determine total protein content and the activities of guaiacol peroxidase (GPX), ascorbate peroxidase (APX), and polyphenol oxidase (PPO).

#### 4.8.2. Quantification of Total Protein

Protein concentration was measured using a microplate reader and the Bradford assay. One 0.5 mL aliquot of the crude extract was thawed on ice. Then, 200 µL of Bradford reagent [59] was added to each well of a 96-well plate, followed by 10 µL of the extract. After gentle pipetting, the plates were incubated at room temperature for 20 min, and the absorbance was recorded at 595 nm. Protein content was determined from a standard curve generated with bovine serum albumin (BSA).

#### 4.8.3. PPO Activity Assay

PPO activity was measured at 25 °C using a modified version of the method by Kar and Mishra (1976) [60] with a microplate reader [38]. Reactions (315 µL) in 96-well plates comprised 50 mM potassium phosphate buffer (pH 6.8), 50 mM pyrogallol, and 10 µL enzyme extract. Absorbance at 420 nm was recorded every 40 s for 4 min using a microplate reader. PPO activity (µmol purpurogallin min^−1^ mg^−1^ protein) was calculated using df = 15.3, ε = 2.47 mM^−1^ cm^−1^, and a 1 cm path length.

#### 4.8.4. GPX Activity Assay

GPX activity was measured at 25 °C using the method of Chance and Maehly [61] with slight modifications. Reactions (200 µL) contained 50 mM potassium phosphate buffer (pH 7.0), 20 mM guaiacol, and 10 mM H_2_O_2_. Enzyme extract (10 µL) was added, and H_2_O_2_ initiated reactions. Absorbance at 470 nm was recorded every 15 s for 3 min. Blanks without enzyme and controls without H_2_O_2_ were included for background correction. Enzyme activity was expressed as ΔA_470_ min^−1^ mg^−1^ protein.

#### 4.8.5. APX Activity Assay

APX activity was measured using the method of Ranieri et al. [62] with slight modifications. Reactions (200 µL) contained 50 mM potassium phosphate buffer (pH 7.0), 0.5 mM L-ascorbic acid, 0.1 mM EDTA, and 0.1 mM H_2_O_2_. Enzyme extract (10 µL) was added, and H_2_O_2_ initiated the reactions. Absorbance at 290 nm was measured every 25 s over 5 min using a double-beam spectrophotometer (Alpha 1900S). Blanks without enzyme and controls without H_2_O_2_ were included for background correction. APX activity was expressed as ΔA290 min^−1^ mg^−1^ protein.

### 4.9. Methanolic Extraction for Antioxidant Assays

For antioxidant analysis, 0.1 g of oven-dried hairy root tissue was ground in a mortar and pestle and transferred to a 2 mL microcentrifuge tube. Under a fume hood, 1 mL of 80% methanol was added, and the tubes were incubated in a water bath at 37 °C for 3 h. The tubes were vortexed every 5 min to improve the extraction process. After extraction, the tubes were centrifuged at 15,000× *g* for 20 min at 4 °C. The supernatant (methanolic extract) was carefully transferred to labeled 1.5 mL tubes and stored at 4 °C until further analysis [63,64].

### 4.10. DPPH Radical Scavenging Activity Assay

To prepare the DPPH solution, about 0.002 g of DPPH powder was dissolved in 50 mL of absolute methanol (100%). According to prior optimization, the most effective concentration of the methanolic extract was found to be 0.125 mM. For each reaction, 1 µL of methanolic extract, 7 µL of 80% methanol, and 272 µL of the DPPH solution were added to the wells of a 96-well microplate. All steps were performed in the dark.

The plates were incubated for 20 min at room temperature, and the absorbance was measured at 517 nm using a microplate reader, with methanol used as the blank. The radical scavenging activity (% inhibition) of each extract was calculated using the following equation [65]:

$$\%I\;=\;(A_{Control}\;-\;A_{Sample})/A_{Control}\;\times \;100$$

### 4.11. Determination of Rosmarinic Acid Content in Hairy Root Samples of D. kotschyi

In this study, the quantification of rosmarinic acid was conducted using a high-performance liquid chromatography (HPLC) system (KNAUER) equipped with a gradient pump and a UV detector set at 330 nm. Separation was performed using a C18 column (25 cm × 4.6 mm, 5 µm particle size). Before extraction, the hairy root samples were dried with a freeze dryer. The freeze-drying process was conducted at a pressure of 270 Pa and a temperature of approximately 60 °C for 48 h. Dried samples were stored and transported in containers containing silica gel for further processing and extraction before HPLC injection.

### 4.12. Data Analysis

The effects of elicitors on rosmarinic acid content in *D. kotschyi* and the activity of antioxidant enzymes were examined at two time points (24 and 48 h) using a factorial experiment based on a completely randomized design (CRD) with three replicates. The elicitor factor was tested at three levels: control, nano titanium dioxide (200 and 400 mg L^−1^), and yeast extract (200 mg L^−1^). Data analysis was performed using SAS software (version 9.4, SAS Institute, Cary, NC, USA), and mean comparisons were made using Duncan’s multiple range test at the 5% probability level.

## 5. Conclusions

This study developed an optimized protocol for inducing and cultivating hairy roots in *D. kotschyi*, offering a practical platform for increased production of secondary metabolites. Initial optimization steps, including explant selection and transformation efficiency, were streamlined to support stable root induction. Among elicitation strategies, treatment with yeast extract (200 mg L^−1^) notably increased the accumulation of rosmarinic acid, antioxidant activity, and protein content. Conversely, higher concentrations of TiO_2_ NPs induced oxidative stress, leading to lower metabolite levels. Overall, yeast extract proved to be a highly effective biotic elicitor for enhancing rosmarinic acid biosynthesis, highlighting its potential for use in biotechnological production systems based on hairy root cultures (Figure S1).

## Figures and Tables

**Table 1:** Primer sequences used in this study.

Gene	Primer ID	Sequence (5′ → 3′)	Primer Length (nt)	Expected Amplicon Size (bp)
*robB*	*RolB* F	5′-GTTCTCGCGAGAAGATGCA 3′	20 bp	bp 780
	*RolB* R	5′-CAGTTTCGCATCTTGACAG-3′	20 bp	
*virD*	*virD F*	5′-ATGCCCGATCGAGCTCAAGT-3′	20 bp	bp 338
	*virD* R	5′-CCTGACCCAAACATCTCGGCT-3′	20 bp	

**Table 2:** Concentrations and exposure times of elicitors used in suspension cultures of *D. kotschyi*.

Elicitor	Applied Concentration	Exposure Time (h)
Yeast extract	200 mg L^−1^	24, 48
TiO_2_	200 mg L^−1^; 400 mg L^−1^	24, 48

## Data Availability

The datasets generated and/or analyzed during this study are available from the corresponding author upon reasonable request. No publicly accessible datasets were used or created in this research. All relevant data supporting the findings are included within the manuscript.

## Supplementary Materials

The following supporting information can be downloaded at: https://www.mdpi.com/article/10.3390/plants14172809/s1, Table S1. Analysis of variance on the effect of sample age and type on hairy root induction in *D. kotschyi*. Table S2. Analysis of variance of the effect of L-arginine on hairy root induction in *D. kotschyi*. Table S3. Analysis of variance (ANOVA) for total protein, DPPH activity, and antioxidant enzymes. Table S4. Analysis of variance of rosmarinic acid. Notes: ns, *, and ** indicate non-significant differences, and significant differences at the 5% and 1% probability levels, respectively. Figure S1. Yeast extract and TiO_2_ NPs markedly enhanced rosmarinic acid accumulation, antioxidant activity, and protein content in hairy root cultures.
