# Supplementary material for: Influence of Biotic and Abiotic Elicitors on Rosmarinic Acid Accumulation in Hairy Root Cultures of Dracocephalum kotschyi Boiss
Source: Plants (Basel). 2025 Sep 8;14(17):2809. doi: 10.3390/plants14172809 (PMC12431348; doi:10.3390/plants14172809)
Supplement: Supplementary file 1 [file plants-14-02809-s001.zip › plants-3818630-supplementary.pdf]

plants

MDPI

Article

Stimulation of Hairy Roots by Rosmarinic Acid Accumulation and Antioxidant in *Dracocephalum kotschyi* Boiss. Using Biotic and Abiotic Elicitors

Hoda Sadat Kiani [1], Manijeh Sabokdast [1*] and Beata Dedicova [2,*]

1 Department of Agronomy and Plant Breeding, College of Agriculture and Natural Resources, University of Tehran, Iran, P.O. Box 4111, 31587-11167 Karaj, Iran; h.kiani@ut.ac.ir (HSK); sabokdast@ut.ac.ir (MS)

2 Department of Plant Breeding, Swedish University of Agricultural Sciences (SLU) Alnarp, Sundsvägen 10, P.O. Box 190, SE -234 22 Lomma, Sweden; beata.dedicova@slu.se (BD)

* Correspondence: sabokdast@ut.ac.ir, beata.dedicova@slu.se

Title

Supplementary Tables for Hairy Root Induction and Antioxidant Analysis in *D.kotschyi.*

Table S1. Analysis of variance on the effect of sample age and type on hairy root induction in *D.kotschyi*

Source of Variation	df	Mean square (MS)
Sample age	2	2680.22**
Error	6	72.66
CV (%)	-	9.18
Sample Type	3	2168.64**
Error	8	18.52
CV (%)	-	5.96

ns, *, and ** indicate non-significant differences, and significant differences at the 5% and 1% probability levels, respectively.

Table S2. Analysis of variance of the effect of L-arginine on hairy root induction in D.kotschyi.

Source of Variation	df	Mean square (MS)
Sample	3	3204.5**
Experimental Error	8	14.2
CV (%)	-	11.5

ns, *, and ** indicate non-significant differences, and significant differences at the 5% and 1% probability levels, respectively.

Table S3. Analysis of variance (ANOVA) for total protein, DPPH activity, and antioxidant enzymes.

Source of Variation	df	Mean square (MS)				
		Total protein	DPPH	GPX	PPO	APX
Elicitor	3	0.138^{**}	35.404^{**}	0.671^{**}	0.000197^{**}	0.0046^{**}
Time	1	0.016^{ns}	16.608^{**}	0.232^{**}	0.000034^{**}	0.0055^{**}
Elicitor × Time	3	0.094^{**}	$15/577^{**}$	2.367^{**}	0.000037^{**}	0.0019^{**}
Experimental Error	16	0.0094	0.0177	0.0053	0.00000060	0.000066
Coefficient of Variation (%)	-	12.67	2.19	5.83	2.74	8.20

ns, *, and ** indicate non-significant differences, and significant differences at the 5% and 1% probability levels, respectively.

Table S4. Analysis of variance of rosmarinic acid.

Source of Variation	df	Mean square (MS)
Elicitor	3	10.66^{**}
Time	1	5.34^{**}
Elicitor × Time	3	1.08^{**}
Experimental Error	16	0.14
Coefficient of Variation (%)	-	2.61

ns, *, and ** indicate no statistically significant difference at the 5% and 1% levels, respectively.

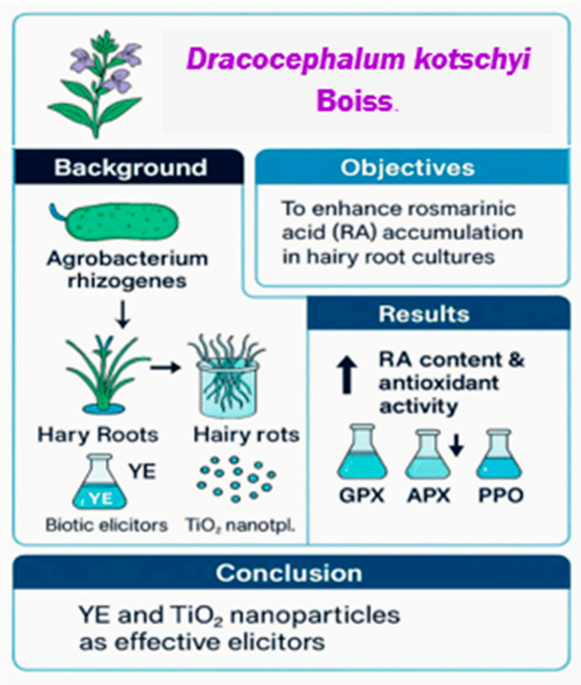

Figure S1. Yeast extract and TiO_2 NPs markedly enhanced rosmarinic acid accumulation, antioxidant activity, and protein content in hairy root cultures.
